# Current Stimulation of the Midbrain Nucleus in Pigeons for Avian Flight Control

**DOI:** 10.3390/mi12070788

**Published:** 2021-06-30

**Authors:** Jungwoo Jang, Changhoon Baek, Sunhyo Kim, Tae-Kyeong Lee, Gwang-Jin Choi, Shinyong Shim, Seunghyeon Yun, Younginha Jung, Chae-Eun Lee, Seunghyung Ko, Kangmoon Seo, Jong-Mo Seo, Moo-Ho Won, Sung J. Kim, Yoon-Kyu Song

**Affiliations:** 1 Graduate School of Convergence Science and Technology, Seoul National University, Seoul 08826, Korea; jungwoojang@snu.ac.kr (J.J.); younginha@snu.ac.kr (Y.J.); celee1025@snu.ac.kr (C.-E.L.); hanre@snu.ac.kr (S.K.); 2 Biomimetic Robot Research Center, Seoul National University, Seoul 08826, Korea; chbaek@snu.ac.kr; 3 Department of Veterinary Clinical Science, Seoul National University, Seoul 08826, Korea; kiemsh@snu.ac.kr (S.K.); kmseo@snu.ac.kr (K.S.); 4 Department of Biomedical Science, Research Institute for Bioscience and Biotechnology, Hallym University, Chuncheon 24252, Korea; tk-lee@hallym.ac.kr; 5 Department of Electrical and Computer Engineering, Seoul National University, Seoul 08826, Korea; rhanrhan100@snu.ac.kr (G.-J.C.); simsinyong@gmail.com (S.S.); shy5812@gmail.com (S.Y.); callme@snu.ac.kr (J.-M.S.); kimsj@snu.ac.kr (S.J.K.); 6 Department of Neurobiology, School of Medicine, Kangwon National University, Chuncheon 24341, Korea; mhwon@kangwon.ac.kr

**Keywords:** pigeon, stereotactic surgery, bird flight, current stimulation, 3D motion tracking

## Abstract

A number of research attempts to understand and modulate sensory and motor skills that are beyond the capability of humans have been underway. They have mainly been expounded in rodent models, where numerous reports of controlling movement to reach target locations by brain stimulation have been achieved. However, in the case of birds, although basic research on movement control has been conducted, the brain nuclei that are triggering these movements have yet to be established. In order to fully control flight navigation in birds, the basic central nervous system involved in flight behavior should be understood comprehensively, and functional maps of the birds’ brains to study the possibility of flight control need to be clarified. Here, we established a stable stereotactic surgery to implant multi-wire electrode arrays and electrically stimulated several nuclei of the pigeon’s brain. A multi-channel electrode array and a wireless stimulation system were implanted in thirteen pigeons. The pigeons’ flight trajectories on electrical stimulation of the cerebral nuclei were monitored and analyzed by a 3D motion tracking program to evaluate the behavioral change, and the exact stimulation site in the brain was confirmed by the postmortem histological examination. Among them, five pigeons were able to induce right and left body turns by stimulating the nuclei of the tractus occipito-mesencephalicus (OM), nucleus taeniae (TN), or nucleus rotundus (RT); the nuclei of tractus septo-mesencephalicus (TSM) or archistriatum ventrale (AV) were stimulated to induce flight aviation for flapping and take-off with five pigeons.

## 1. Introduction

Electrical microstimulations in the brain, as well as peripheral nerves, enable sensory or motor rehabilitation [1,2,3,4,5,6,7,8,9] such as cochlear implants for the deaf [10,11,12,13,14], deep brain stimulators for Parkinsonism or essential tremors [15,16], and cardiac pacemakers for arrhythmia [17]. Neural engineering for non-human application is also under investigation, such as ‘animal-bots’ for the safe and fast screening of dangerous or disastrous situations.

After the establishment of the brain stereotactic surgery technique using the stereotactic frame in birds [18], researchers have focused on flight control by electrically stimulating the brain of freely moving pigeons [19,20,21,22,23,24,25,26,27,28]. A Swedish research group [29,30] inserted nichrome or platinum-iridium electrodes into the pigeon’s forebrain and applied currents between 0.05−1 mA to induce specific behaviors in the subject, such as concentration, nodding, flapping, avoidance, and head-turning. Furthermore, the nerve nucleus of the pigeon’s midbrain was electrically stimulated to induce flight control movements, such as forward step, take-off, and body rotation [22]. In a follow-up study, periodic stimuli were delivered to a pigeon’s brain, causing right or left body turns, [22,28] while a global positioning system (GPS) was attached to analyze its location and confirm induction of the target behaviors. Since then, multi-array microelectrodes that are inserted at once and shorten the time of surgery succeeded in inducing pigeons’ body turns and forward walks on the ground using a wireless stimulation module [19,31]. Such a system in combination with GPS tracking succeeded in controlling left and right aviation [27]. One group recorded brain stimulation experiments in pigeons with a ceiling-mounted camera and analyzed the flight paths quantitatively by changing the stimulation parameters and comparing the trajectories without stimulation [24].

According to the research group, response to the electrical stimulation of the specific cerebral nuclei varies; formatio reticularis medialis mesencephali (FRM) [22] or dorsalis intermedius ventralis anterior (DIVA) [19,27] for lateral body turn in flying pigeons; nucleus intercollicularis (ICo) for take-off [22,28] or walking forward [25]. So far, studies on functional brain maps for controlling pigeon flight are not well established, and it is still impossible to control a pigeon’s flight path by stimulating the previously reported nuclei.

In this study, cerebral nuclei targeted in previous reports were evaluated and the nuclei for controlling take-off and lateral body turn in a reproducible manner were investigated. For this purpose, stereotactic neurosurgery under general anesthesia, wireless neuro-stimulation system, and video-based flight analysis in pigeons were established.

## 2. Materials and Methods

### 2.1. Animals and Ethics Statement

Pigeons, Columba livia, aged 2−6 years old, and about 500 g in weight were selected regardless of gender. Stereotactic brain surgery with microelectrodes was performed under general anesthesia, and the microelectrodes were implanted by stereotactic brain surgery. Pigeons that underwent electrode implantation recovered in cages exclusively for birds with controlled illumination, and food and water supply. The normal consumption of food and changes in behavior were monitored.

The day after surgery, behavioral and locomotive changes on electrical brain stimulation were investigated without anesthesia. Electrical stimulation was limited to 15 times per stimulation, and there were at least 3-min intervals between the stimulations. The pigeons were euthanized soon after the stimulation experiment; under general anesthesia, cardiac arrest was induced by the intravenous injection of potassium chloride under electrocardiographic monitoring. All procedures mentioned above, from surgical implantation to euthanasia, were done under the approval of the Institutional Animal Care and Use Committees (IACUC) of Seoul National University (SNU-170622-2).

### 2.2. Polymer-Based Deep Brain Electrode Arrays

Because deeply seated motor nuclei of the brain were investigated as stimulation targets for rotation and flight in pigeons, penetrating depth probe electrodes were designed. Liquid crystal polymer (LCP)-based fully implantable brain stimulation system was fabricated and used in this experiment because LCP has excellent biocompatibility and low moisture permeability, and can be fabricated into a multi-channel depth probe electrode array, while its flexibility reduces the risk of electrode fracture [23,32,33]. The fabricated LCP electrode had 10 mm shanks with two stimulation sites per shank. These 100 μm × 300 μm stimulation openings were separated by 1 mm to compensate for the possible mismatch between the brain atlas and the individual entities. Shanks were designed to be thin and flexible, enabling stable fixation through calvarium [18], and merged as a single external connector. The electrical impedance of the electrode was 9.53 kΩ at 1 kHz and a charge storage capacity of 155 mC/cm^2^, adequate for electrical brain stimulation.

### 2.3. Implantation Procedure

Because continuous intravenous infusion or repetitive intramuscular injection of the anesthetic agent is not appropriate for the hour-long operations, inhalation anesthesia was adopted. Isoflurane (Ifran Solution^®^; Hana Pharm., Korea) was used as an inhalation anesthetic and intubation was used for maintaining inhalation anesthesia with 1.8−2.2% of isoflurane. Capnography and respiratory sounds were monitored. Meloxicam (Metacam injection^®^, Boehringer Ingelheim, Germany) was used for analgesic and anti-inflammatory purposes, and cephazolin (Cephazolin injection^®^, Chong Kun Dang Pharm., Korea) as prophylactic antibiotics. To avoid hypothermia during surgery, the cloacal temperature was monitored, and a blanket with a warming unit (3TM Bair Hugger^TM^ Patient Warming Unit, Model 505) was applied.

**Figure 1:**
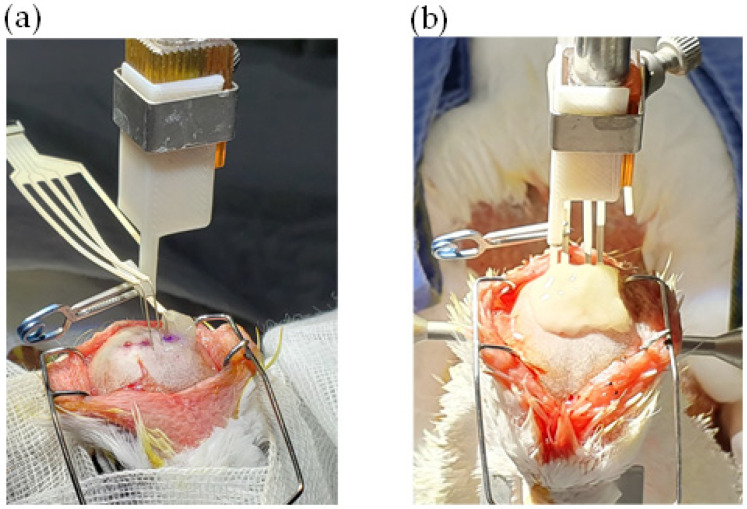
Stereotactic implantation of an electrode array to pigeon’s brain. There are four shanks for active electrodes and one shank for a reference electrode. (**a**) Shanks of the multielectrode array were introduced one by one into the target nuclei, and (**b**) each shank was fixed to the calvarium by glass ionomer dental cement.

The implantation of the electrodes was performed in a small animal stereotactic instrument (model 900LS, with model 918 pigeon adaptor, David Kopf Instruments, Tujunga, CA, USA). After scalp incision, burr holes were made over calvarium by dental needle at exact points of the implantation. According to the pigeon brain atlas [18], shanks of the multielectrode array were introduced one by one into the target nuclei, and each shank was fixed to the calvaria by glass ionomer dental cement (Fuji II LC, GC Corp., Tokyo, Japan) as shown in Figure 1. The scalp was sutured with 4-0 Monocryl^®^ (poliglecaprone 25, Ethicon US, LLC, Cincinnati, OH, USA) around the electrode array [32].

### 2.4. Implantable Wireless Current Stimulator

A handheld, wireless neural stimulation controller and the implantable neurostimulator developed by the authors were used for the experiment [26,32,34]. The external controller was composed of a microcontroller (SPARTAN3A, Xilinx, San Jose, CA, USA) and a Zigbee communication module (CC2530, Texas Instruments, Dallas, TX, USA). The neural stimulation controller visually indexed when the electrical stimulation was successfully transmitted to the implanted stimulation device and checked the battery level of the implanted device. The implanted wireless stimulation system consisted of a custom 16-channel stimulation microcontroller unit (MCU), Zigbee communication module, an inductive coil for power delivery, and a data receiving antenna. The stimulation current waveform could be adjusted by receiving pulse width modulation (PWM) data with a 2–5 MHz carrier frequency as input. The pulse was modified in the range of 40.7–452.3 Hz for the frequency, 10.1–636.3 μs for the duration, and 0–10.23 mA for the amplitude. The size of the designed wireless stimulation device [34] for implant was 29 mm × 26 mm × 8 mm, and was hermetically packaged with LCP.

### 2.5. 3D Motion Tracking

**Figure 2:**
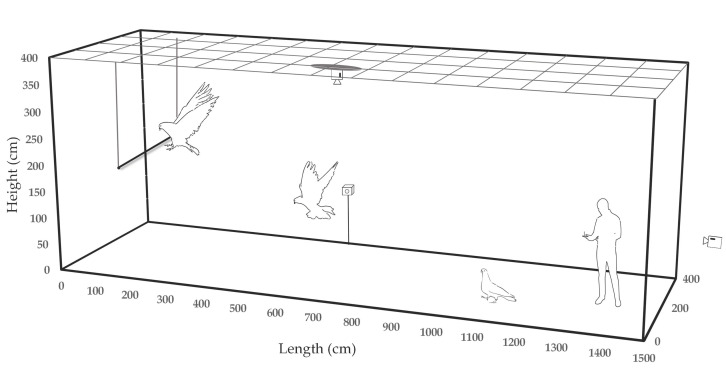
Diagram of 3D motion tracking. Cameras were orthogonally placed to acquire the pigeon’s flight trajectory in Cartesian coordinate.

Flight trajectory, velocity, and acceleration data during electrical stimulation of the pigeon’s brain were acquired by two wide-angle action cameras (Hero4, GoPro Inc, San Mateo, CA, USA) and the DeepLabCut 3D (Mathis Lab, Geneva, Switzerland) motion capture program (Figure 2). One camera as a top-down view and the other as a side view simultaneously recorded the video of the pigeon’s flight to acquire an orthogonal coordinate of the flight path. Metric scale rulers were placed on the floor and on the wall taken by the camera to calculate the real-world Cartesian coordinates from video clips. In two video clips, pigeons were manually marked on several key frames, and the DeepLabCut 3D program estimated the pigeon’s position in the remaining sequential frames.

Indoor walking, body turn, flapping or ultra-short range flying on electrical stimulation of the brain was evaluated in the multidisciplinary laboratory for animal experiments at the SNU veterinary hospital, and outdoor, short-range flying on electrical stimulation of the brain was evaluated in the aviary of the same hospital, the size of which is about 4 m in width, 15 m in length, and 3.5 m in height.

### 2.6. Histological Evaluation

Because the midbrain motor nuclei are located so close to each other and the size of the cerebrum varies minutely between pigeons, micro-scale deviation of the electrode position on the target site may occur. To accurately localize the position of the stimulating electrode in the pigeon brain, postmortem histological evaluation was done.

After euthanasia, the brain was harvested and was fixed in 4% paraformaldehyde, and then infiltrated in 30% sucrose solution. Cryosection was prepared by sliding microtome (SM2010 R, Leica Biosystems, Nussloch, Germany) equipped with a freezing platform (BFS-40MP, Physitemp Instruments Inc., New Jersey, NJ, USA). Cresyl violet (CV) stain was done to examine the morphology of neural cells and the pattern of neuronal distribution, and the actual coordinates of the electrode insertion were determined by comparing the CV-stained section and an atlas of the pigeon brain [18,35].

## 3. Results

### 3.1. Without Electrical Brain Stimulation

**Figure 3:**
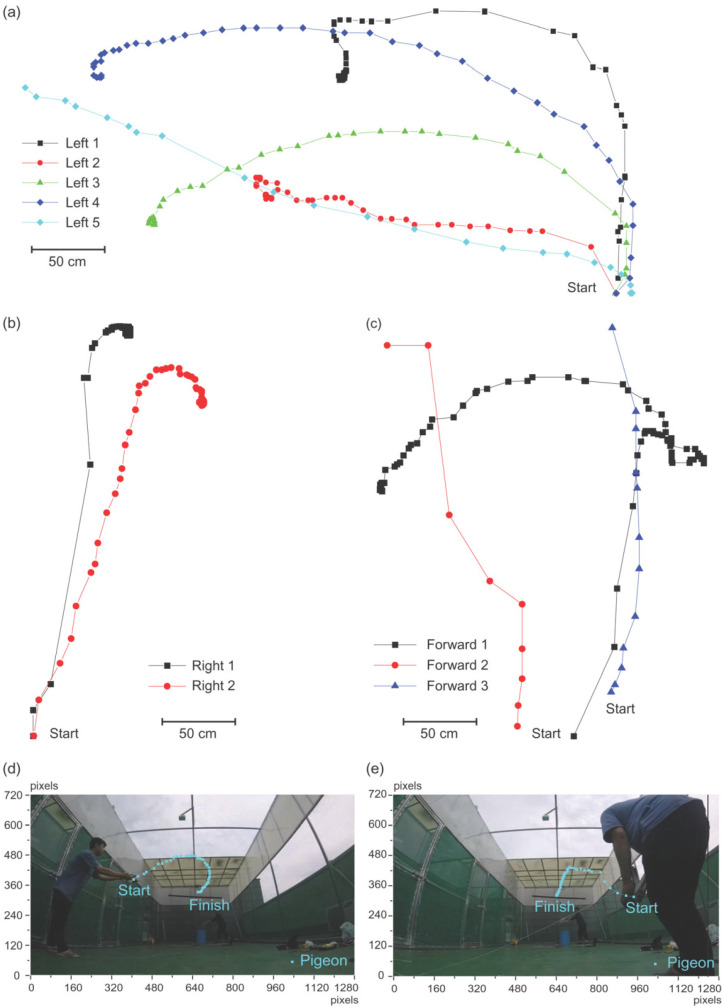
Sample trajectories of free flying pigeons without electrical brain stimulation. Indoor trajectories of a pigeon grouped as (**a**) leftward, (**b**) rightward, and (**c**) forward flight. In the aviary, when pigeons flown from the (**d**) left or (**e**) right, landed on a perch at the end of their flight regardless of the flying path.

First, the flight trajectory without electrical brain stimulation was observed. The pigeon flew and sat on an obstacle or landed during indoor evaluation in the multidisciplinary laboratory for animal experiments. The flying path and the final landing were random because there were many objects and obstacles in the room. For example, a pigeon flew to the left five times, twice to the right, and forward three times among ten attempts (Figure 3a–c). In the aviary, the pigeons landed on a perch at the end of their flight regardless of the flying path, and they usually flew straight toward the perch (Figure 3d,e).

### 3.2. Behavioral Change on Electrical Midbrain Stimulation

**Figure 4:**
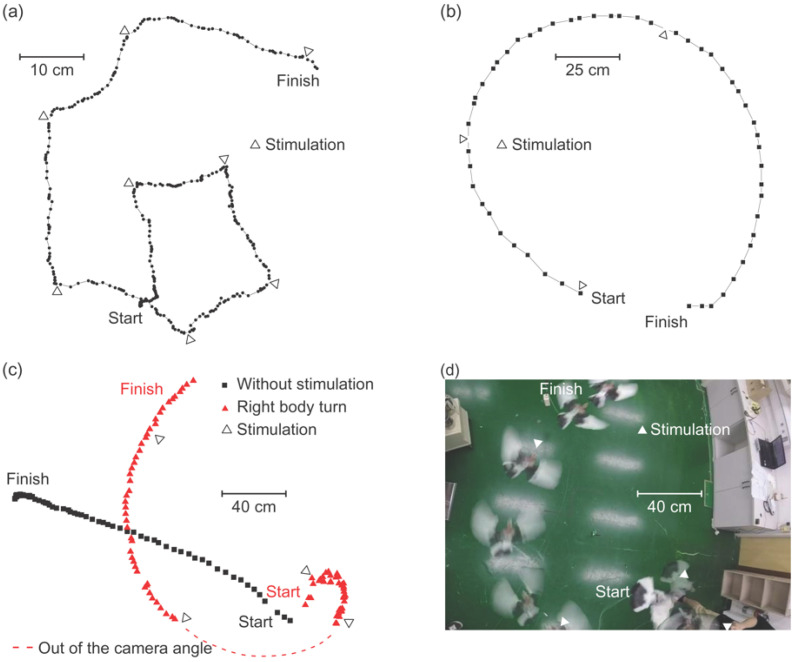
Behavioral changes on electrical current stimulation of the midbrain nucleus. A pigeon showed (**a**) right body turn on the ground and (**b**) rightward turn during flight during electrical stimulation. (**c**) Without stimulation, the pigeon flew forward (black square dots), and with stimulation, flew rightward (red triangular dots). (**d**) stroboscopic image of the rightward flight of the pigeon by electrical stimulation.

Four pigeons showed right body turn on the ground by the electrical current stimulation of the midbrain nuclei as shown in Figure 4, which was confirmed by the repetitive experiment described in Section 2.1. Rightward deviation of the flight path was induced during the flight by electrical current stimulation (Figure 4b). Parameters of the stimulation were 226 Hz, 1mA for 160 μs for the right body turn on the ground, and 226 Hz, 2 mA for 160 μs for the rightward turn during flight. Among these four, the position of the electrode could be localized in three cases by the postmortem histological evaluation, and these were the OM tractus occipitomesencephalicus (OM) or nucleus taeniae (TN).

**Figure 5:**
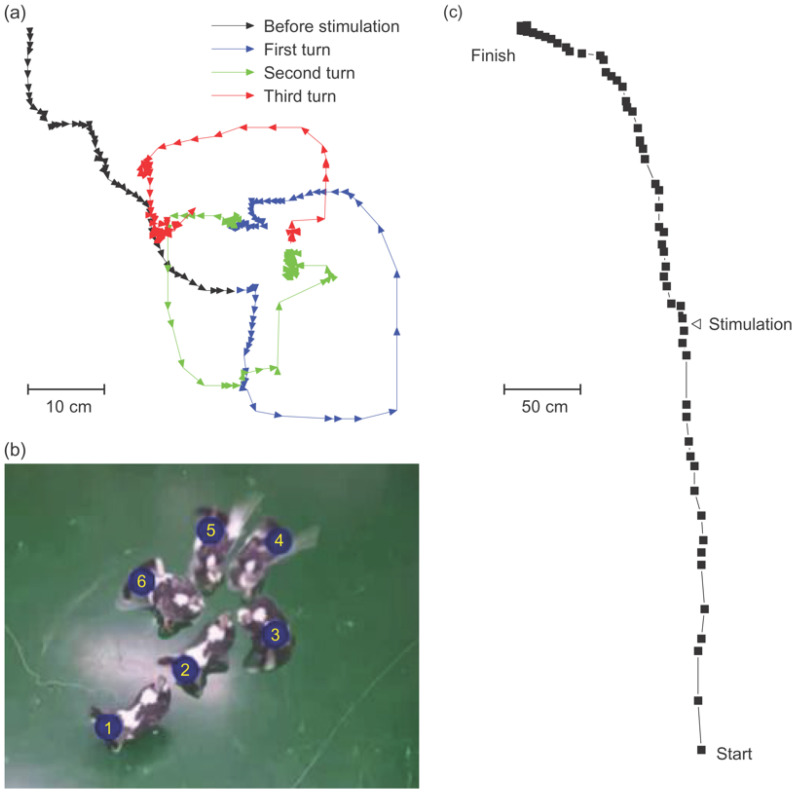
Behavioral changes on electrical current stimulation of the midbrain nucleus. A pigeon with a marker in its tail showed (**a**) the counterclockwise, leftward body turn on the ground during stimulation. It showed three consecutive turns in a single stimulation session. (**b**) stroboscopic image of the leftward body turn on the ground. (**c**) The trajectory of the leftward flight in the outdoor aviary was induced by electrical stimulation.

Eight pigeons showed counterclockwise, left body turns on the ground by the electrical current stimulation of the midbrain nuclei. During the stimulation, the trajectory of the tail made a circle as shown in Figure 5a, which was confirmed by the repetitive experiment described in Section 2.1. Leftward deviation of the flight path was induced during the flight by electrical current stimulation. Parameters of the stimulation were 226 Hz, 1.6 mA for 80 μs for the left body turn on the ground, and was 226 Hz, 5 mA for 80 μs for the leftward turn during flight. On postmortem histological evaluation, the position of the electrode was confirmed as the necleus rotundus (RT) in two cases.

**Figure 6:**
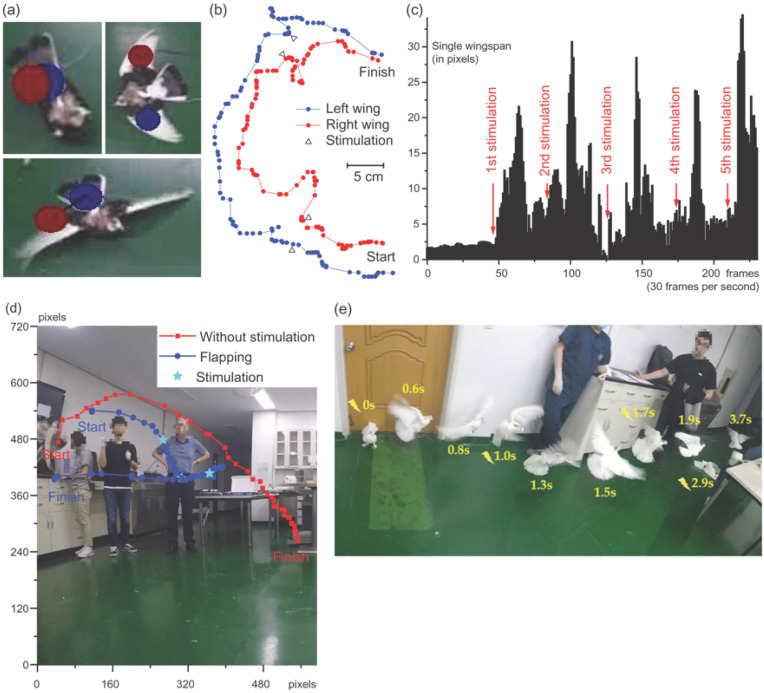
Behavioral changes on electrical current stimulation of the midbrain nucleus. A pigeon that has markers selected to its wings confirmed the flapping of wings and the take-off. (**a**) Representative image of the blue and red markers selected to either both wings, or a body and a wing, for the AI-based 3D motion tracking program. (**b**) Extension and retraction of wings during and after the take-off. (**c**) The distance between the body and one of the wings. (**d**) Comparison of flight under nucleus stimulation and without stimulation. (**e**) stroboscopic image of walking pigeon’s take-off during stimulation.

Eight pigeons showed flapping of wings from the ground when electrically stimulated, four of which took-off at higher stimulation parameters. This motion was confirmed by the repetitive experiment described in Section 2.1. The trajectories of both the left and the right wings, which were tagged as designated tracking points, showed that the pigeon did flap its wings, and the characteristics of flapping were analyzed by the change of the Cartesian coordinates of the tracking points as shown in Figure 6b. The parameters of the stimulation for the flapping of wings from the ground were 226 Hz, 1.5 mA, for 160 μs.

**Figure 7:**
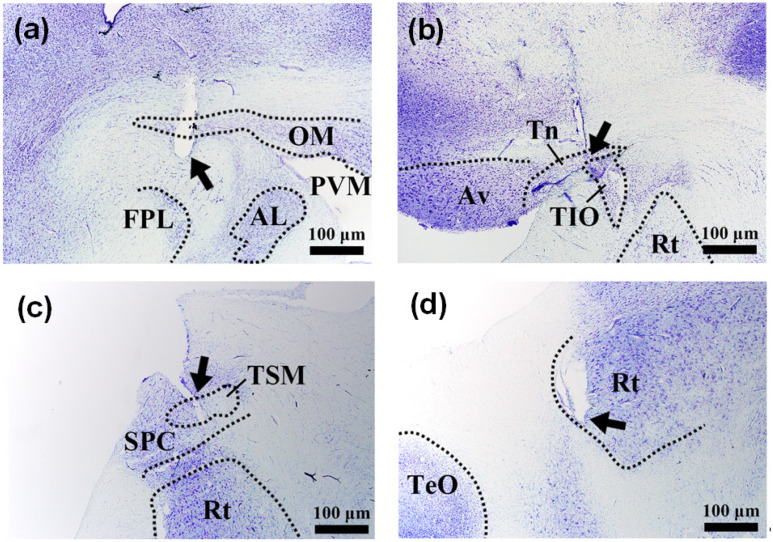
Histology of pigeon brain to confirm electrode insertion coordinates. The pigeon (**a**) turned to the right on tractus occipito-mesencephalicus (OM) stimulation, (**b**) flew to the left on nucleus taeniae (TN) stimulation, (**c**) flapped both wings or took-off on tractus septo-mesencephalicus (TSM) stimulation, and (**d**) turned to the left on nucleus rotundus (RT) stimulation.

Taking-off could be analyzed by tracking the body from the side. The parameters of the stimulation for the take-off were 226 Hz, 4 mA, for 160 μs. The pigeons immediately landed once the stimulation was discontinued, however, with repeated stimulation, they maintained their altitude for up to several seconds (Figure 6d). On postmortem histological evaluation, the position of the electrode was confirmed as the tractus septo-mesencephalicus (TSM) or archistriatum ventral (Av) (Figure 7c). Notably, one of the pigeons showed the same take-off response from a much lower stimulation amplitude of 2 mA, which was later discovered that the electrode was mainly stimulating the Av.

### 3.3. Histological Evaluation

Postmortem histological evaluation was done in all thirteen pigeons as described in Section 2.6, and the successful localization with functional matching could be achieved in eight cases. Even though the orientation of the harvested brain in the microtome was carefully fitted according to the alignment during the stereotactic surgery, perfect reproduction of the orientation was challenging. As a result, the discriminative location of the electrode in the midbrain could not be localized accurately in five cases.

Thirteen pigeons were implanted with the LCP microelectrode array through stereotactic brain surgery. Three or four active electrodes were implanted per pigeon to induce left or right body turn, wing flapping, and forward walking. Nine pigeons were implanted with one of the electrodes intended to induce the right body turn, four of which showed the intended response. Histological analysis was performed on three of the four pigeons that showed the intended response, which confirmed that either OM or Tn was stimulated. Twelve pigeons were implanted with one of the electrodes intended to induce the left body turn, eight of which showed the intended response. Histological analysis was conducted on two of eight pigeons, and it was confirmed that the RT was stimulated. Finally, twelve pigeons were implanted with one of the electrodes intended to induce the wing flapping, eight of which flapped their wings, and took off when introduced to a stronger stimulation. Histological analysis was conducted on five of the eight pigeons, and it was confirmed that either TSM or Av was stimulated. The histology results are shown in Figure 7.

## 4. Discussion

In 2015, the central nerve nuclei related to take-off, forward walk, and lateral body turns were proposed [22]. Cai et al. [22], confirmed that body turning is induced when the ICo nucleus or locus ceruleus (LoC) is stimulated, and lateral body turning was triggered upon applying current stimulation to the FRM or nucleus vestibularis dorsolateralis (VeDL) nucleus in the fixed head of a lightly anesthetized pigeon. In 2018, a wireless current stimulator, a global positioning system IC and a memory card were attached to trained pigeons flying to a certain target places using the pigeons’ homing instinct and the left or right FRM nucleus was stimulated during stable flight to induce lateral body turns [28]. After the pigeons arrived at the trained place, the flight trajectory was extracted from the data stored on the memory card to verify that the desired flight aviation was induced when electrical stimulation was applied. Others stimulated the DIVA nucleus to induce a lateral body turn in pigeons driven by fear [19,31]. For example, when stimulating the left DIVA nucleus, a pigeon feels like it is being touched in the right side and turns the body to the left in repulsion. [19,25], In order to walk along a tape attached in a straight line to the ground, electrical stimulation was applied to the DIVA nucleus, causing a lateral body to turn and the periaqueductal gray (PAG) nucleus, which induces forward walking. In addition, Huai et al., Yang et al. [19,31] and Wang et al. [28], verified that stimulating the left or right DIVA nucleus could control lateral body turning in trained pigeons. However, we found that lateral body aviation can be induced by applying current to the RT, OM, and TN nuclei through a fully implantable wireless pigeon flight control stimulation system. Although we have not yet analyzed the neurological mechanism triggering flight, it is interesting that we could induce the same locomotion in different coordinates. Cai et al. and Wang et al. [22,28] proposed the ICo nucleus can control pigeon’s wing flapping and take-off while another research group [25] suggested that ICo is more suitable for inducing walk forward locomotion rather than take-off. Thus, two research groups have different interpretations of flight locomotion when stimulating the ICo nucleus. We succeeded in inducing wing flapping and take-off by stimulating the ICo nerve nucleus, but it was confirmed that when the electrode was inserted a little deeper into the ICo nucleus coordinates, and the distance was close to the FRM nucleus, when a strong current stimulus was applied, lateral body turns were induced rather than wing flapping. Additionally, we confirmed the forward walking locomotion by stimulating the straum griseum centrale (SGC). However, no further stimulation experiments were conducted because of the low reproducibility of SGC nucleus stimulation. We further found that stimulating TSM and Av nuclei could induce flapping and take-off.

As shown in the histological examination, we had a pigeon with electrodes inserted in coordinates capable of simultaneously stimulating the OM nucleus, inducing lateral body turn, and the TSM nucleus, that can induce take-off or wing flapping. When stimulating its brain, we could infer that the newly discovered nerve nucleus was suitable for controlling the pigeon flight, as it turned to the right after flapping on the ground. Table 1 shows the key nuclei of pigeon flight control inducing the same movements but with different coordinates to those previously reported [19,22,24,25,27,28,31]. Compared with FRM, which is the nucleus related to pigeon body turn and ICo which induces flapping, the larger TSM and OM nuclei have the advantage of being able to insert electrodes with more coordinates.

Compared to the stimulation parameters from previous research [22,24,25], we applied stronger electrical stimulation to the pigeons’ nuclei. When we first implanted the complete stimulation system, we needed to apply a strong current stimulation to control pigeon locomotion on the ground. In addition, even during the first wireless flight control at the outside aviary, we could only induce the desired flight movements through the maximum stimulation conditions the system could produce. With continued optimization of the wireless stimulation system and a more sophisticated electrode insertion technique, we could control pigeons’ flight with <1 mA. However, this stimulus amplitude is still quite high compared to previous research. Cai et al. [22] propose that 14.8 μA to 30.3 μA of stimulation current can induce pigeon locomotion in head-fixed animals under anesthesia, but there was no mention of stimulus conditions during free flight. Another study [25] reported that it was possible to induce forward walking and lateral body turns from the ground with a 50–120 μA amplitude. In terms of stimulation current amplitude, other groups [22,25] seem to have succeeded in controlling pigeon locomotion with smaller currents than ours, but comparing the current density based on the stimulation electrode pad open size, we induced flight aviation with a similar or smaller electric charge density. The comparison of stimulation current density for each research group is shown in Table 2.

The study had some limitations. Unlike rodents [36,37,38], pigeons have a long flight distance and a large space is essential for in vivo stimulation experiments. All experiment were done in the animal ethics guidelines-approved aviary of the College of Veterinary Medicine building at SNU. Because the flying speed of a pigeon is 50 to 70 km/h [39], the flight time in this aviary was less than 5 s per experiment. This limited the experiment to single-electrode stimulation instead of multi-electrode (multiple sites) stimulation for the reliable interpretation of the flight trajectory change. The other is that even though the LCP-based neural interface is well known for its good biocompatibility in vitro, long-term in vivo biocompatibility is not yet proved in birds, thus long-term, repetitive electrical stimulation could not be done in this experiment.

Future work will include the long-term in vivo evaluation of the implantable neural interface in the bird, multiple site stimulation for inducing complex behavioral change, and the long-term, repetitive electrical midbrain stimulation.

## 5. Conclusions

Investigations are underway to control flight aviation such as take-off and lateral body turn. We stimulated previously reported brain nuclei [19,22,24,25,27,28,31] related to pigeon flight to verify the accuracy of our stereotactic surgery technique and further applied electrical stimulation in other nuclei to examine related flight control regions. We extracted the pigeon flight trajectory through a 3D motion capture program which proved that we could induce specific flight patterns by current stimulation. With this approach, we identified new nuclei that can induce the same flight movements as previously described but with lower current density. Brain biopsies confirmed that the same locomotion was induced when the same nucleus was stimulated, and the stimulation electrode was successfully inserted at the same brain coordinates.

Studies of neurological brain mapping or induction of certain behaviors in birds have been attempted for decades, but the results from a few research groups are insufficient compared to rodents. In this study, we discovered some fundamental components of pigeon locomotion, the brain nuclei involved in the lateral turn and take-off actions. Also, the anesthesia, stereotactic surgery, and flight analysis technologies that have been established during this research are expected to become steppingstones for future neuroscience research as well as neuroengineering applications in birds.

## Figures and Tables

**Table 1:** Functions of cerebral nuclei were confirmed by the behavioral evaluation and the histological examination.

Behavioral Change	Stimulation Parameter	Number of Pigeons Confirmed by		Midbrain Nucleus Confirmed on Histological Examination
		Trajectory Evaluation	Histological Examination *	
Right body turn during flight	2 mA 226 Hz 160 μs	4	3	OM (tractus occipito-mesencephalicus), TN (nucleus taeniae)
Left body turn during flight	5 mA 226 Hz 80 μs	8	2	RT (nucleus rotundus)
Wing flapping	1.5 mA 226 Hz 160 μs	8	5	TSM (tractus septo-mesencephalicus) Av (archistriatum ventrale)
Take-off	4 mA 226 Hz 160 μs	4	4	TSM (tractus septo-mesencephalicus) Av (archistriatum ventrale) **

* Cases only confirmed by trajectory evaluation were double-checked by the histological examination. ** One case showed a low current threshold of stimulation as 2 mA at Av only.

**Table 2:** Comparison of the stimulation parameters in this work with those in the previous research.

	2015 [22]	2018 [25]	2019 [24]	This work
Amplitude	14.8 μA–30.3 μA	50 μA–120 μA	60 μA–450 μA	1 mA–5 mA
Current density (μA/μm^2^)	0.04–0.09	0.07−0.17	-	0.03-0.17
Electrode	Stainless steel	Four pairs of resin-coated stainless-steel microelectrode	Stainless steel, Teflon insulation	LCP microelectrode array
Pad open size (μm^2^)	314	706	-	30,000
Target nucleus for lateral body turn	FRM, VeDL	DIVA	FRM	OM, TN, RT
Target nucleus for take-off	ICo, LLd, FRM, Loc	-	-	TSM, Av
Flying	×	×	○	○

## References

[1] Aravanis A.M., Wang L.P., Zhang F., Meltzer L.A., Mogri M.Z., Schneider M.B., Deisseroth K. (2007). An optical neural interface: In vivo control of rodent motor cortex with integrated fiberoptic and optogenetic technology. J. Neural Eng..

[2] Charkhkar H., Shell C.E., Marasco P.D., Pinault G.J., Tyler D.J., Triolo R.J. (2018). High-density peripheral nerve cuffs restore natural sensation to individuals with lower-limb amputations. J. Neural Eng..

[3] Chouinard P.A., Van der Werf Y.D., Leonard G., Paus T. (2003). Modulating neural networks with transcranial magnetic stimulation applied over the dorsal premotor and primary motor cortices. J. Neurophysiol..

[4] Christie B.P., Charkhkar H., Shell C.E., Marasco P.D., Tyler D.J., Triolo R.J. (2019). Visual inputs and postural manipulations affect the location of somatosensory percepts elicited by electrical stimulation. Sci. Rep..

[5] Dhillon G.S., Horch K.W. (2005). Direct neural sensory feedback and control of a prosthetic arm. IEEE Trans. Neur. Syst. Rehabil. Eng..

[6] Kim H., Kim S., Sim N.S., Pasquinelli C., Thielscher A., Lee J.H., Lee H.J. (2019). Miniature ultrasound ring array transducers for transcranial ultrasound neuromodulation of freely-moving small animals. Brain Stimul..

[7] Lee S.H., Jeong S.H., Jun S.B., Kim S.J., Park T.H. (2009). Enhancement of cellular olfactory signal by electrical stimulation. Electrophoresis.

[8] Peterson E.J., Tyler D.J. (2014). Motor neuron activation in peripheral nerves using infrared neural stimulation. J. Neural Eng..

[9] Reilly J.P., Freeman V.T., Larkin W.D. (1985). Sensory Effects of Transient Electrical-Stimulation—Evaluation with a Neuroelectric Model. IEEE Trans. Biomed. Eng..

[10] An S.K., Park S.I., Jun S.B., Lee C.J., Byun K.M., Sung J.H., Wilson B.S., Rebscher S.J., Oh S.H., Kim S.J. (2007). Design for a simplified cochlear implant system. IEEE Trans Biomed. Eng..

[11] Gwon T.M., Min K.S., Kim J.H., Oh S.H., Lee H.S., Park M.H., Kim S.J. (2015). Fabrication and evaluation of an improved polymer-based cochlear electrode array for atraumatic insertion. Biomed. Microdevices.

[12] Park J.H., Kim J.H., Song Y.K., Jung Y., Hur S., Kim W., Kim S.J. (2013). Design of an Analog Front End for a Bio-Inspired Auditory Sensor of a Novel Totally Implantable Cochlear Implant. Sens. Mater..

[13] Ulusan H., Chamanian S., Ilik B., Muhtaroglu A., Kulah H. (2019). Fully Implantable Cochlear Implant Interface Electronics with 51.2-mu W Front-End Circuit. IEEE Trans. Very Large Scale Integr. Syst..

[14] Ulusan H., Chamanian S., Zorlu O., Muhtaroglu A., Kulah H. Neural stimulation interface with ultra-low power signal conditioning circuit for fully-implantable cochlear implants. Proceedings of the 2017 IEEE Biomedical Circuit and Systems Conference (BioCAS).

[15] Fregni F., Boggio P.S., Santos M.C., Lima M., Vieira A.L., Rigonatti S.P., Silva M.T.A., Barbosa E.R., Nitsche M.A., Pascual-Leone A. (2006). Noninvasive cortical stimulation with transcranial direct current stimulation in Parkinson’ s disease. Mov. Disord..

[16] Panikar D., Kishore A. (2003). Deep brain stimulation for Parkinson’ s disease. Neurol. India.

[17] Abiri P., Abiri A., Packard R.R.S., Ding Y.C., Yousefi A., Ma J.G., Bersohn M., Nguyen K.L., Markovic D., Moloudi S. (2017). Inductively powered wireless pacing via a miniature pacemaker and remote stimulation control system. Sci. Rep..

[18] Karten H.J., Hodos W. (1967). A Stereotaxic Atlas of the Brain of the Pigeon (Columbia livia).

[19] Huai R.T., Yang J.Q., Wang H. (2016). The robo-pigeon based on the multiple brain regions synchronization implanted microelectrodes. Bioengineered.

[20] Wylie D.R., Frost B.J. (1993). Responses of Pigeon Vestibulocerebellar Neurons to Optokinetic Stimulation. 2. The 3-Dimensional Reference Frame of Rotation Neurons in the Flocculus. J. Neurophysiol..

[21] Wylie D.R., Kripalani T., Frost B.J. (1993). Responses of Pigeon Vestibulocerebellar Neurons to Optokinetic Stimulation.1. Functional-Organization of Neurons Discriminating between Translational and Rotational Visual Flow. J. Neurophysiol..

[22] Cai L., Dai Z.D., Wang W.B., Wang H., Tang Y.Z. (2015). Modulating Motor Behaviors by Electrical Stimulation of Specific Nuclei in Pigeons. J. Bionic. Eng..

[23] Choi G.J., Jang J., Kang S., Shim S., Baek C., Kim B., Park Y., Kim S., Jung Y., Seo K. Locomotion Control of Pigeons using Polymer-based Deep Brain Electrodes. Proceedings of the 2018 Annual International Conference of the IEEE Engineering in Medicine and Biology Society 2018.

[24] Zhao K., Wan H., Shang Z.G., Liu X.Y., Liu L. (2019). Intracortical microstimulation parameters modulate flight behavior in pigeon. J. Integr. Neurosci..

[25] Wang H., Yang J.Q., Lv C.Z., Huai R.T., Li Y.X. (2018). Intercollicular nucleus electric stimulation encoded “walk forward” commands in pigeons. Anim. Biol..

[26] Shim S., Yun S., Kim S., Choi G.J., Baek C., Jang J., Jung Y., Sung J., Park J.H., Seo K. (2020). A handheld neural stimulation controller for avian navigation guided by remote control. Biomed. Mater. Eng..

[27] Yang J.Q., Huai R.T., Wang H., Li W.Y., Wang Z.G., Sui M., Su X.C. (2017). Global Positioning System-Based Stimulation for Robo-Pigeons in Open Space. Front. Neurorobotics.

[28] Wang H., Li J.J., Cai L., Wang C., Shi A.J. (2018). Flight control of robo-pigeon using a neural stimulation algorithm. J. Integr. Neurosci..

[29] Akerman B. (1966). Behavioural Effects of Electrical Stimulation in the Forebrain of the Pigeon. I. Reproductive. Brill Behav..

[30] Akerman B. (1966). Behavioural effects of electrical stimulation in the forebrain of the pigeon. II. Protective behaviour. Brill Behav..

[31] Yang J., Huai R., Wang H., Lv C., Su X. (2015). A robo-pigeon based on an innovative multi-mode telestimulation system. Biomed. Mater. Eng..

[32] Baek C., Kim S., Jang J.W., Jung Y., Choi G.J., Shim S., Yun S., Seo K., Song Y.K., Kim S.J. (2020). Investigation of stereotactic surgery for avian brain stimulation by a fully implanted wireless system. Neurosurg. Focus.

[33] Lee S.E., Jun S.B., Lee H.J., Kim J., Lee S.W., Im C., Shin H.C., Chang J.W., Kim S.J. (2012). A Flexible Depth Probe Using Liquid Crystal Polymer. IEEE Trans. Biomed. Eng..

[34] Yun S., Koh C.S., Jeong J., Seo J., Ahn S.H., Choi G.J., Shim S., Shin J., Jung H.H., Chang J.W. (2019). Remote-Controlled Fully Implantable Neural Stimulator for Freely Moving Small Animal. Electronics.

[35] Lee T.K., Park J.H., Ahn J.H., Park Y.E., Park C.W., Lee J.C., Choi J.H., Hwang I.K., Kim S., Shim J. (2019). Parvalbumin-immunoreactive cells in the olfactory bulb of the pigeon: Comparison with the rat. Anat. Histol. Embryol..

[36] Sun C., Zheng N., Zhang X., Chen W., Zheng X. An Automatic Control Model for Rat-robot. Proceedings of the 2011 Annual International Conference of the IEEE Engineering in Medicine and Biology Society.

[37] Huai R., Yang J., Wang H., Su X. A new robo-animals navigation method guided by the remote control. Proceedings of the BMEI 2009: 2nd International Conference on Biomedical Engineering and Informatics.

[38] Wang H., Huai R., Yang J., Su X. A wireless remote control system applied in roborat research based on Brain-Computer. Proceedings of the 2012 IEEE International Conference on Computer Science and Automation Engineering.

[39] Schiffner I., Fuhrmann P., Reimann J., Wiltschko R. (2018). Behavioural traits of individual homing pigeons, *Columba livia* f. *domestica*, in their homing flights. PLoS ONE.

